# Complexity of Plasmodium falciparum infection and genetic variations associated with differences in parasite clearance time in two Malian villages

**DOI:** 10.21203/rs.3.rs-3083860/v1

**Published:** 2023-06-26

**Authors:** Sekou Sissoko, Aminatou Kone, Antoine Dara, Mary Aigbiremo Oboh, Bakary Fofana, Cheick O. Sangare, Demba Dembele, Aboubecrine Sedhigh Haidara, Nouhoum Diallo, Sekou Toure, Kadidia Haidara, Kassim Sanogo, Ogobara K. Doumbo, Amed Ouattar, Alfred Amambua-Ngwa, Abdoulaye A. Djimde

**Affiliations:** Malaria Research and Training Center, Faculty of Pharmacy, University of Sciences, Techniques and Technologies of Bamako; Malaria Research and Training Center, Faculty of Pharmacy, University of Sciences, Techniques and Technologies of Bamako; Malaria Research and Training Center, Faculty of Pharmacy, University of Sciences, Techniques and Technologies of Bamako; Medical Research Council Unit, The Gambia at London School of Hygiene and Tropical; Malaria Research and Training Center, Faculty of Pharmacy, University of Sciences, Techniques and Technologies of Bamako; Malaria Research and Training Center, Faculty of Pharmacy, University of Sciences, Techniques and Technologies of Bamako; Malaria Research and Training Center, Faculty of Pharmacy, University of Sciences, Techniques and Technologies of Bamako; Malaria Research and Training Center, Faculty of Pharmacy, University of Sciences, Techniques and Technologies of Bamako; Malaria Research and Training Center, Faculty of Pharmacy, University of Sciences, Techniques and Technologies of Bamako; Malaria Research and Training Center, Faculty of Pharmacy, University of Sciences, Techniques and Technologies of Bamako; Malaria Research and Training Center, Faculty of Pharmacy, University of Sciences, Techniques and Technologies of Bamako; Malaria Research and Training Center, Faculty of Pharmacy, University of Sciences, Techniques and Technologies of Bamako; Malaria Research and Training Center, Faculty of Pharmacy, University of Sciences, Techniques and Technologies of Bamako; University of Maryland Baltimore, Baltimore, MD; Medical Research Council Unit, The Gambia at London School of Hygiene and Tropical; Malaria Research and Training Center, Faculty of Pharmacy, University of Sciences, Techniques and Technologies of Bamako

## Abstract

**Background:**

Effective approaches to fight against malaria include disease prevention, an early diagnosis of malaria cases, and rapid management of confirmed cases by treatment with effective antimalarials. Artemisinin-based combination therapies are first-line treatments for uncomplicated malaria in endemic areas. However, cases of resistance to artemisinin have already been described in South-East Asia resulting in prolonged parasite clearance time after treatment. In Mali, though mutations in the K13 gene associated with delayed clearance in Asia are absent, a significant difference in parasite clearance time following treatment with artesunate was observed between two malaria endemic sites, Bougoula-Hameau and Faladje. Hypothetically, differences in complexity of *Plasmodium falciparum* infections may be accounted for this difference. Hence, the aims of this study were to assess the complexity of infection (COI) and genetic diversity of *P. falciparum* parasites during malaria treatment in Bougoula-Hameau and Faladje in Mali.

**Methods:**

Thirty (30) patients per village were randomly selected from 221 patients enrolled in a prospective artesunate monotherapy study conducted in Faladje and Bougoula-Hameau in 2016. All parasitemic blood samples of patients from enrollment to last positive slide were retained to assess malaria parasite COI and polymorphisms. DNA were extracted with a Qiagen kit and *Pfcsp* and *Pfama1* encoding gene were amplified by nested PCR and sequenced using the Illumina platform. The parasite clearance time (PCT) was determined using the parasite clearance estimator of Worldwide Antimarial Resistance Network (WWARN). Data were analyzed with R^®^.

**Results:**

The median number of genetically distinct parasite clones was similar at enrollment, 7 (IQR of 5–9) in Faladje and 6 (IQR of 4–10) in Bougoula-Hameau (p-value = 0.1). On the first day after treatment initiation, the COI was higher in Faladje (6; CI:4–8) than in Bougoula-Hameau (4; CI:4–6) with a p-value =0. 02. Overall, COI was high with higher PCT. Finally, there was a low genetic diversity between Faladje and Bougoula-Hameau

**Conclusion:**

This study demonstrated that the difference in PCT observed between the two villages could be due to differences in the complexity of infection of these two villages.

## 1. Introduction

Malaria is a febrile and hemolyzing erythrocytopathy due to the presence and the development in the liver and then in the blood of a hematozoa of the genus *Plasmodium*. It is transmitted to humans by the bite of a female Anopheles mosquito, although, other means of transmission such as transfusion and fetal-maternal route exist [1, 2]. Six species of *Plasmodium: P. falciparum, P. malariae, P.ovale wallikeri, P.ovale cutisi, P. vivax and P. Knowlesi* infect humans, *P. falciparum being the most lethal species*.

Despite the substantial progress made in malaria treatment and prevention, it remains a serious public health challenge in Africa. In 2021, there were an estimated 241 million cases of malaria worldwide, with 95% occurring in Africa [3]. WHO reported 627 000 deaths in the world including 64% in WHO African region[3]. According to the Local System of Sanitary Information (SLIS) of Malian ministry of Health, malaria accounted for 32% of disease and 22% of deaths in 2018 [4]; the incidence of uncomplicated malaria was highest in children 1–4-year-old (158.3 cases per 1000) followed by 0–11 months (145 cases per 1000) [4].

Artemisinin-based combination therapies (ACT) are the recommended first line treatment of uncomplicated malaria in endemic countries [5]. In Mali, combinations of Artemether-Lumefantrine (AL) and Artesunate-Amodiaquine (ASAQ) are used for the treatment of uncomplicated malaria [6]. The advent of ACTs raised great hope for malaria control as they allow rapid and effective clearance of malaria parasite from infected individuals. Combined with other malaria control strategies such as intermittent preventive treatment in pregnancy (IPTp), indoor residual spraying (IRS), use of insecticide-treated nets (ITNs) and seasonal malaria chemoprevention (SMC), they allowed steady decreased in malaria deaths from 896 000 in 2000 to 558 000 in 2019 [3].

However, there have been concerns of reduced artemisinin efficacy in Southeast Asia [7] since 2009. Thus, it’s possible for these resistant parasites to develop or spread to other parts of the world especially in sub-Saharan Africa. Artemisinin resistance is defined as a prolong parasite clearance time after artemisinin treatment and/or associated with mutations in the *P. falciparum* kelch 13 (*Pfkelch13*) propeller domain gene [8]. Emergence of *P. falciparum* with high survival rates by ring survival assay associated with the *Pfkelch13* A675V mutation has already been reported in Uganda in 2018 [9]. This is in addition to the observation of *Pfkelch13* R561H mutation associated with delayed parasite clearance after AL treatment in Rwanda [10]. These together are raising fears of emergence and spread of artemisinin resistance across Africa, hampering current gains in reducing malaria morbidity and mortality.

Following the detection of artemisinin resistance cases in Southeast Asia, a prospective therapeutic efficacy study of artesunate conducted in Mali in 2010–2011 reported no evidence of delayed parasite clearance [11]. Although mutations in *Pfkelch13* gene were described in samples collected during the study, they were not associated with artemisinin resistance [12]. In view of monitoring the efficacy of artemisinin in Mali, a similar prospective therapeutic efficacy study of artesunate was repeated in two villages of Mali during the 2015–2016 transmission season [13]. The findings suggest a significant difference in parasite clearance half-life between the two villages [13]. Moreover, the *Pfkelch13* A578S mutation was observed in one sample (1/98) in Bougoula-Hameau while no such mutation was found in Faladje (N = 118) [13]. This difference in parasite clearance rates between the two villages could not be explained by mutations in the *Pfkelch13* gene. The discrepancy may probably be due to other factors such as immunity or complexity of infection (COI).

The complexity of malaria infection is defined as the number of genetically distinct parasite variants co-infecting a single host, which is an important indicator of malaria epidemiology [14]. Malaria transmission intensity can affect the complexity of infection which can modify clinical outcomes of malaria disease [15]. Complexity of infection and genetic diversity have been described as negatively correlated with antimalarial drug efficacy [16].

We hypothesized that the complexity of infection and genetic diversity contribute to differences in sensitivity of *P. falciparum* infections to Artesunate monotherapy in Mali. To test our hypothesis, we assessed the dynamic of complexity of infection and the genetic diversity of *P. falciparum* in two malaria endemic villages of Mali during treatment with artesunate monotherapy.

## 2. Materials and Methods

### 2.1. In vivo artesunate efficacy study

A prospective therapeutic efficacy study of artesunate was conducted from October 2015 to March 2016 in patients who were at least 6 months of age or older and diagnosed with uncomplicated *P. falciparum* malaria in Bougoula-Hameau and Faladje. We enrolled 121 participants in Faladje and 100 in Bougoula-Hameau. The protocol of this study was approved by the Ethics Committee of the Faculty of Medicine, Pharmacy, and Stomatology and written informed consent was obtained from the parent or guardian of each child before inclusion. Participants were hospitalized and treated for 7 days with artesunate monotherapy and were until all malaria symptoms and parasitemia were resolved. They were then followed-up for 28 days. Brielfly, four (4) mg / kg of artesunate was administered (Asunate Denk, Denk Pharma, Munich, Germany) on day 0 then 2 mg / kg from day 1 to day 7. Slides and dry blood spots were collected every 8 hours until three consecutive slides were negative. Additional blood specimens were collected on days 2, 3, 7, 14, 21 and 28. Blood smears were screened for parasitemia by two trained microscopists according to WHO standard procedures [17].

### 2.2. Sampling

We randomly selected 30 patients per site using the “sample” function of the R package. A total of 259 time points from the 60 patients were sampled and used for sequencing. These time points represented all the positive dried blood spots (DBSs) from enrollment to the last positive slide.

### 2.3. Molecular confirmation of *P. falciparum*, amplification, and sequencing of Pfama1 and Pfcsp

*P. falciparum* DNA was extracted from DBS using QIAGEN kit according to the manufacturer’s instructions (Qiagen^®^). After DNA extraction a qPCR assay was used to amplify the varATS gene to verify the presence of *P. falciparum* parasite DNA in the extracts [18]. We used amplicon deep sequencing of *Pfama1* and *Pfcsp* to investigate the complexity of infection and parasite genetic diversity in infections from Bougoula-Hameau and Faladje. These genes were amplified by nested PCR using two sets of primers, *Pfama1*-F1 (GAA GTT CAT GGT TCA GGT ATA AG); *Pfama1*-R1 (GTA TGG TTT TTC CAT CAG AAC TGG) and *Pfcsp*-F1(GTC GGA ATT CAT GAT GAG AAA ATT AGC TATT); *Pfcsp*-R1 (CTA ATT AAG GAA CAA GAA GG) to amplify the outer regions of *Pfama1* (1503 bp) and *Pfcsp* (1194 bp) respectively. Two others set of primers *Pfama1*-NF (GAT GCT GAA GTA GCT GGA ACTC); *Pfama1*-NR (GTG ATG CTC TTT TTT CTT CCC CCC) and *Pfcsp*-NF (TCG TCA AAC ACA AGG GTT CT); *Pfcsp*-NR (ACG ACA TTA AAC ACA CTG GAA CA) were used for nested PCR to amplify 1432 bp of *Pfama1* and 1058 bp of *Pfcsp*. For primary and nested PCR, *Pfama1* and *Pfcsp* were amplified under similar cycling conditions: 98°C for 1 min, followed by 35 cycles with 45 s of denaturation at 98°C, annealing at 63°C for 45 s, and elongation at 72°C for 1 min. After 35 cycles, a final elongation step at 72°C for 5 min. After amplification, the PCR product was visualized on 1,2% agarose gel. PCR products were quantified by Qubit and normalized prior to pooling amplicons of the two genes for each sample. Pooled amplicons were used for library prepared using NEBNext^®^ Ultra^™^ II kit following the manufacturer’s instructions. Libraries were quantified on Qubit and fragment sizes visualized on the Agilent 2200 TapeStation system. Library concentrations were used to pool indexed samples and sequenced (paired end) on Illumina Miseq platform.

### 2.4. Data workflow and Statistics analysis

**Figure 1:**
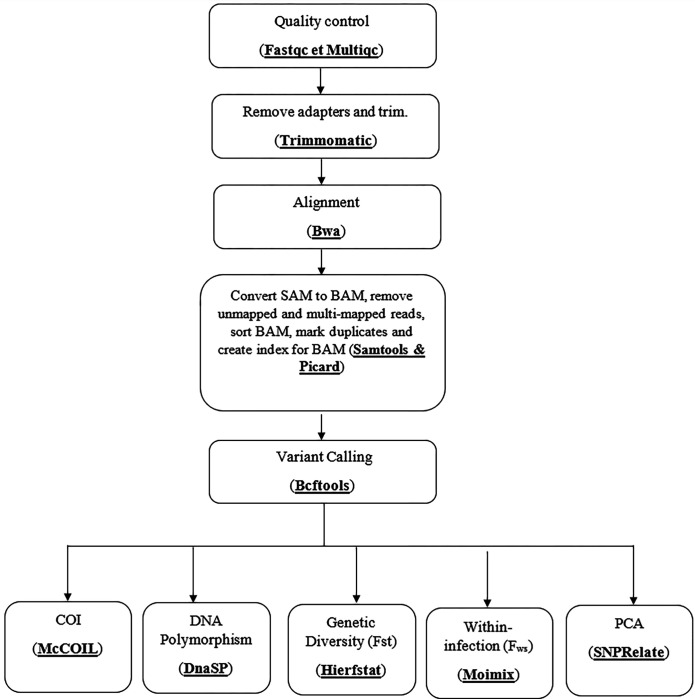
Bioinformatic analysis pipeline applied on amplicon sequencing data. Sequence reads quality was assessed using Fastqc and Multiqc. Trimmomatic was then used to remove adapters and trim low-quality nucleotides (Figure 1). Next, reads were aligned to the reference (PF3D7) sequences of each gene using BWA and SAM files were converted into BAM files. During the next step, unmapped and multi-mapped reads were removed, and sorted BAM files marked duplicates and created index for BAM files using samtools and picard (Figure 1). Variant calling was done with bcftools and the vcf file generated was used with different packages of R to generate the complexity of infection based on polymorphism of *Pfama1* and *Pfcsp*, the genetic diversity (Fst), the within-infection fixation indices (*F*_WS_) and run a principal component analysis (PCA analysis) in order to identify structure in the distribution of genetic variation across the two villages at different of follow up time point.

Sequence reads quality was assessed using Fastqc and Multiqc. Reads were aligned to the reference (PF3D7) sequences of each gene using BWA and SAM files generated from this step were converted into BAM files (Fig. 1). During the next step of analysis, unmapped and multi-mapped reads were removed, and sorted BAM files marked duplicates and created index for BAM files using samtools and picard (Fig. 1). Variant calling was done with bcftools and the vcf file generated was used with different packages of R to generate the complexity of infection based on polymorphism of *Pfama1* and *Pfcsp*, the genetic diversity (Fst), the within-infection fixation indices (*F*_WS_) and run a principal component analysis (PCA analysis) in order to identify structure in the distribution of genetic variation across the two villages at different of follow up time point (Fig. 1).

The nucleotide diversity analysis was performed using DnaSP v5. We used Student and Wilcoxon test to compare the complexity of infection and nucleotide diversity within and between populations. Linear regression implemented in ggplot R package were used to assess the correlation between the complexity of infection and parasite density, and *F*_WS_ and parasite clearance time, respectively. The significance level was set at 5%.

## 3. Results

In both villages the number of samples per time point was around 30 except for timepoint H32 in Bougoula-Hameau and Faladje where only respectively 3 and 21 patients had positive slides.

### 3.1. Parasite clearance time.

**Figure 2:**
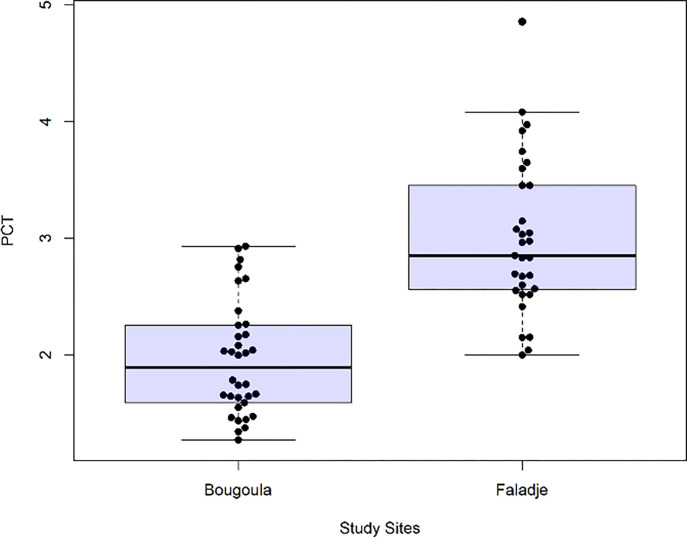
Distribution of parasite clearance Time (PCT) by villages. The panel shows the parasite clearance time calculated using the parasite clearance estimator (PCE). The horizontal lines are medians, and the brackets represent interquartile ranges. Different values of PCT are plotted as circles.

The median of parasite clearance time (PCT) was assessed and compared between the two study sites. An analysis of the difference of PCT between the two villages showed that the median PCT was significantly lower (p-value<0.01) in Bougoula-Hameau (2 hours) compared to Faladje (3 hours) (Figure 2).

### 3.2. Dynamics of complexity of infection

**Figure 3:**
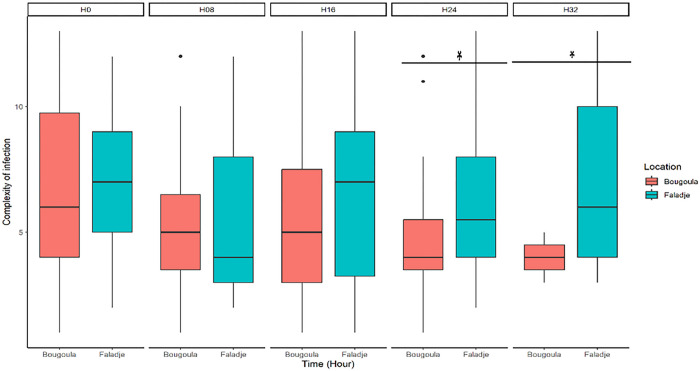
Box plots showing the dynamic of complexity of infection (COI) during malaria treatment with artesunate monotherapy in Bougoula-Hameau and Faladje. The x-axis represents study sites (Bougoula and Faladje) for each time point (H0, H08, H16, H24, H32) while the y-axis corresponds to the COI. The box indicates the IQR (25% and 75%); the thick line within the box represents the median.

There was a decrease in the COI in Bougoula-Hameau during the follow-up time, with samples collected 32 hours post-treatment having the least COI, while those collected before treatment and up to 16 hours post-treatment had the highest COI (Figure 3). Conversely, there was no clear pattern in the evolution of COI over time in Faladje (Figure 3).

There was no difference between the median number of clones described based on *Pfama1* and *Pfcsp* polymorphisms at enrollment in Bougoula-Hameau and Faladje (p-value=0.1). However, the number of clones was higher (p-value = 0.02) in Faladje compared to Bougoula Hameau one day after treatment initiation (Figure 3).

**Figure 4:**
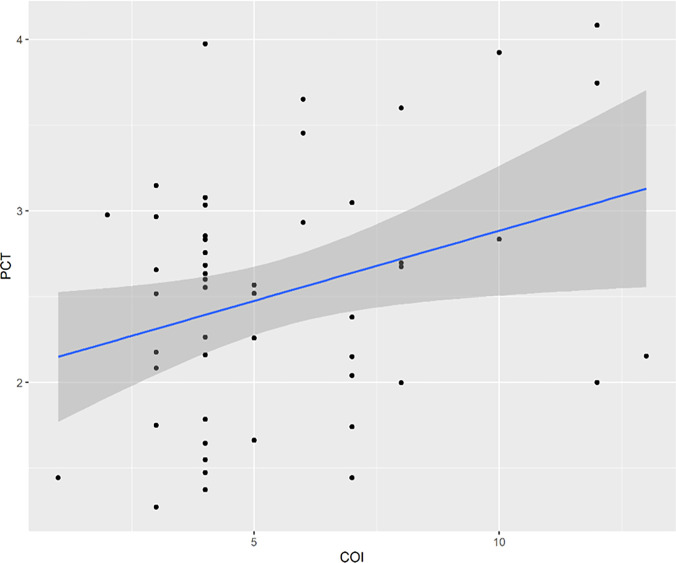
Correlation between COI and parasite clearance time. The x-axis represents F_WS_ index while the y-axis corresponds to the COI. The correlation was statistically significant, p-value=0.02 r = 0.31.

Overall, the complexity of infection was positively associated with the parasite clearance time with the most complex infections having the longest parasite clearance time (Figure 4).

**Figure 5:**
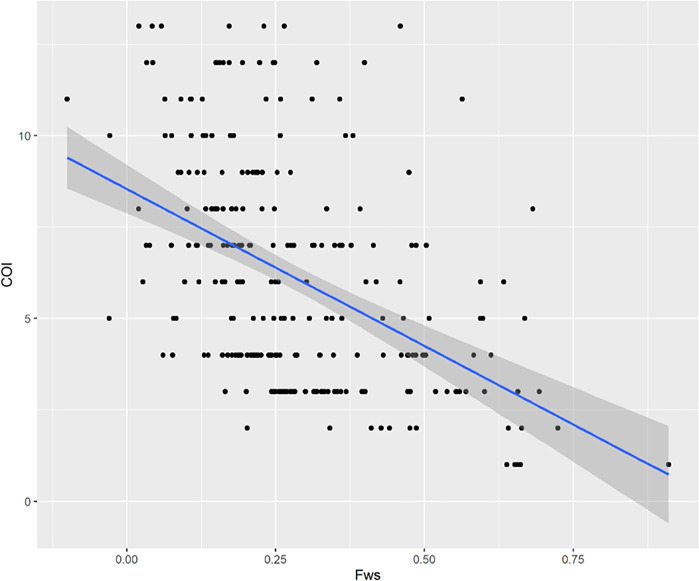
Correlation between F_WS_ and Complexity of infection (COI). The x-axis represents F_WS_ index while the y-axis corresponds to the COI. The correlation was statistically significant, p-value <0.001, r = −0.5.

There was a significant negative correlation between COI and the inbreeding coefficient (*F*_WS_) (p-value <0.001)_,_ with the least complex infections having the highest *F*_WS_ (Figure 5).

### 3.3. Population genetic parameters in Bougoula-Hameau and Faladje.

To assess the effect of artesunate therapy on *in vivo* malaria parasite diversity over the course of treatment, we measured parasites diversity parameters during the follow-up period. The mean nucleotide diversity parameter (π) was lower one day post treatment than at enrollment in Bougoula and Faladje with p-value respectively equal to 0.02 and 0.002 (Table 1). The mean of F_WS_ was already greater in Bougoula-Hameau compared to Faladje at baseline (p-value=0.001) and one day after treatment initiation (p-value=0.03), indicating that the within-host parasite diversity was high in Faladje compared to Bougoula-Hameau (Table 1).

As infection diversity and clearance time were different between the two study sites, we analyzed for possible population substructure in these Malian malaria parasite populations. The population Wright’s differentiation index (F_ST_) between Bougoula-Hameau and Faladje was generally low, with an F_ST_ value of 0.001 at baseline and 0.002 one day after treatment. (Table 1).

**Figure 6:**
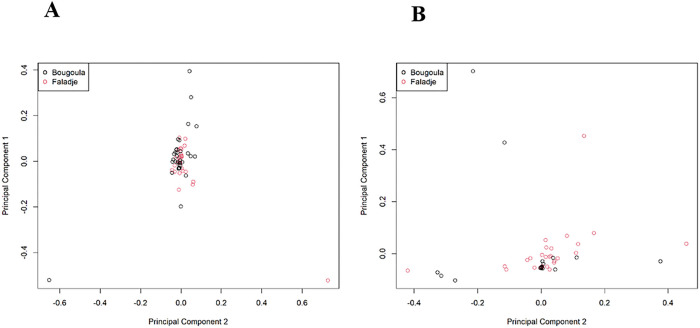
Principal component analysis at baseline (A, left panel) and H24 (B, right panel). At baseline there was no sub-structuring between parasites of Bougoula-Hameau and Faladje. However, the parasites were more similar at baseline than after one day of treatment.

At the initiation of treatment, isolates from each of the sites did not cluster differently as shown on the PCA plot (Figure 6A). A similar pattern was observed for isolates 24 hours after treatment, although isolates from both sites were more dispersed from a scatter plot of dimension 1 and 2 of PCA (Figure 6B).

## 4. Discussions

Malaria treatment with artemisinin-based combination drugs have averted millions of deaths since their introduction almost two decades ago [19, 20]. Reducing malaria mortality depends on their continuous efficacy through monitoring. Here, we used a deep sequencing approach to generate sequence data of two *P. falciparum* polymorphic genes and showed that parasite clearance time was associated with malaria complexity of infections in two Malian villages. This difference in infections complexity could explain the observed difference in parasite clearance time between the two villages following uncomplicated malaria treatment with artesunate in monotherapy. These findings are supported both by analyses of complexity of infections (COI) and within-infection fixation indices (*F*_WS_) between the two study sites.

Our findings suggest a decline in the mean nucleotide diversity parameter (π) from enrollment to 24 hours after initiation of treatment in Bougoula-Hameau, while the pattern was not stable between time points in Faladje. Faladje was characterized with long parasite clearance times associated with more multiple strain (polygenetic) infections. These results suggest that genetic diversity at commencement of treatment may have contributed to the parasite clearance rate, with more complex infection responding slower to drugs. As the parasite populations from the two villages showed no population substructure from Fst and PCA analysis, there is adequate gene flow between the two sites, difference in transmission immunity may also have affected clearance time.

Artemisinin resistance is measured by slow parasite clearance rates. The relevance of this definition and the importance of these phenotypes in Mali where parasitemia is mostly high needs further investigation. These assessments require consideration of the direct effect of artemisinin and its companion drugs, and other factors including sequestration and immunity on drug efficacy. While a study by Lee *et al* [21] supported slower clearance rated for more complex infections, Topazian *et al* [22] have found that low complexity of infection to be rather associated with molecular persistence of *P. falciparum* in Kenya and Tanzania. This is contrary to our findings, although in this case we used artesunate monotherapy instead of an ACT as conducted by Topazian *et al* [22]. On the other hand, hyperimmune malaria serum have been shown to increase the PCT in infected individuals [23], although seroprevalence of malaria antibodies did not explain the shorter clearance time for *P. falciparum* parasites during ACT treatment in Democratic Republic of the Congo [24]. These studies also used combination drugs rather than artesunate monotherapy, and hence did not assess the effect of the artemisinin derived component only. As the biomarkers for differences in clearance time in Mali could be different, this warrants further investigation.

The village with more complex infections, Falaje, had a higher clearance time. Higher diversity and infection complexity reflect the intensity of transmission, with higher transmission driving the acquisition of immunity, thus helping drugs to clear malaria parasites [25, 26]. This is contrary to what was observed for Falaje, which had more complex infections. It is likely that immunity alone was less efficient against complex infections with multiple strains of the parasite in a single infection. Moreover, the biomass of parasite strains in addition to undetermined parasite genetic factors may be playing a role in parasite evasion of drugs and immunity.

The inbreeding coefficient is used to understand malaria parasite population biology as it undergoes gamete formation and fertilization during mosquito lifecycle [27]. Highly complex infections results from high diversity in *P. falciparum* population as observed in high transmission settings, while low transmission settings are characterized by more monoclonal infections [28, 29]. Complex infections remain highly prevalent in Mali but this varies between different geographic areas [30, 31]. At baseline the median number of parasite clones per infection was twice as high compared to previous medians reported respectively in Mali and Burkina Faso [31, 32]. These differences might be due to the epidemiological differences (intensity of transmission and parasite density) of our study sites and variation in the study periods, which can influence malaria transmission and therefore the complexity of the infection [33]. The isolates analyzed here were from 2015–2016, a period when malaria transmission was relatively high in these Mali study sites. These studies where done six years ago, and it is possible that the continued decline in malaria incidence as elimination interventions were implemented might have changes the scenario and response to drugs. However, Mali remains one of the countries with the highest incidence of malaria in West Africa and application of genomic surveillance to monitor drug interventions could help refine elimination strategies.

The novelty and strength of this work is the significant positive association between parasites clearance time (which is used to monitor artemisinin efficacy instead of the adequate clinical and parasitological response) and the complexity of infection and the significant difference between parasite clearance time and complexity of infection between the two villages. In addition, this study used artesunate monotherapy, allows for an unbiased monitoring of the efficacy of an artemisinin derivative, contrary to ACTs where there is interference from partner drug efficacy.

Our study has some limitations. Genetic diversity analyses have been carried out using amplicon deep sequencing of two polymorphic genes (*Pfama1* and *Pfcsp*) instead of whole genome, missing important contributions that may have come from other genes. However, we believe our approach has not significantly impacted on our conclusions. We also did not determine artesunate pharmacokinetics during the *in vivo* efficacy study, which would have allow us determine any differences in plasma drug concentration, that could influence drug efficacy [34].

## 5. Conclusions

This study described a significant difference in complexity of infection between Bougoula-Hameau and Faladje 24 hours after artesunate monotherapy treatment. Complexity of infection positively correlates with the parasite clearance time and could be a significant determinant in treatment efficacy of artemisinin derivatives and ACTs.

## Figures and Tables

**Table 1:** Population genetic statistics of isolates from Bougoula-Hameau and Faladje

Genetic parameters	Sites	H0	H08	H16	H24
Nucleotide diversity(pi) Mean [CI 95%]	Bougoula-Hameau	0.143 [0.099–0.188]	0.082 [0.058–0.106]	0.114 [0.072–0.157]	0.078 [0.056–0.100]
	Faladje	0.157 [0.114–0.200]	0.115 [0.086–0.144]	0.119 [0.080–0.158]	0.104 [0.069–0.139]
Index F_WS_ Mean [CI 95%]	Bougoula-Hameau	0.347 [0.287–0.407]	0.303 [0.238–0.369]	0.339 [0.281–0.398]	0.330 [0.252–0.408]
	Faladje	0.214 [0.164–0.265]	0.320 [0.243–0.398]	0.189 [0.137–0.240]	0.232 [0.187–0.277]
Index F_ST_ Mean [CI 95%]	Bougoula-Hameau Versus Faladje	0.001 [−0.002–0.004]	0.002 [−0.001–0.006]	0.0003 [−0.003–0.004]	0.003 [−0.002–0.007]

H0, H08, H16 and H24: Parasites collected before inclusion, 8 hours, 16 hours, and 24 hours after treatment.
